# Underlying Mechanisms and Candidate Drugs for COVID-19 Based on the Connectivity Map Database

**DOI:** 10.3389/fgene.2020.558557

**Published:** 2020-10-27

**Authors:** Zhonglin Li, Ling Yang

**Affiliations:** Division of Gastroenterology, Union Hospital, Tongji Medical College, Huazhong University of Science and Technology, Wuhan, China

**Keywords:** severe acute respiratory syndrome coronavirus 2, angiotensin-converting enzyme 2, underlying mechanisms, potential drugs, coronavirus disease 2019

## Abstract

**Background:**

The coronavirus disease 2019 (COVID-19) has become a worldwide public health crisis. At present, there are no effective antiviral drugs to treat COVID-19. Although some vaccines have been developed, late-stage clinical trials that allow licensure by regulatory agencies are still needed. Previous reports have indicated that severe acute respiratory syndrome coronavirus 2 (SARS-CoV-2) and SARS-CoV are highly homologous and both use angiotensin-converting enzyme 2 (ACE2) as the receptor to enter cells, and SARS-CoV infection reduces the ACE2 expression in the lung. Therefore, the analysis of genes co-expressed with ACE2 in the lung may uncover the underlying mechanism of COVID-19. Finally, we used the Connectivity map (Cmap) database to search for candidate drugs using transcriptome profiles of patients with COVID-19.

**Method:**

Based on the differentially expressed genes (DEGs), indicated by the expression of RNAs isolated from bronchoalveolar lavage fluid (BALF) cells of patients with COVID-19, we performed functional enrichment analysis and hub gene cluster analysis. Furthermore, we identified genes co-expressed with ACE2 in healthy lung samples and analyzed the significant genes. Additionally, to identify several candidate drugs for the treatment of COVID-19, we queried Cmap using DEGs and genes co-expressed with ACE2.

**Results and Conclusion:**

The up-regulated genes in the BALF cells of patients with COVID-19 are related to viral mRNA translation. The down-regulated genes are related to immune response functions. Genes positively correlated with ACE2 are related to immune defense and those negatively correlated are enriched in synaptic transmission functions. The results reflected prosperous viral proliferation and immune dysfunction in patients. Furthermore, ACE2 may not only mediate viral entrance, but also play an important role in immune defense. By using Cmap with transcriptome profiles of patients with COVID-19, we identified candidate drugs for the treatment of COVID-19, such as amantadine and acyclovir.

## Introduction

The outbreak of SARS-CoV-2 started in 2019, and extended to multiple continents within a month, which has been declared to be a public health emergency of international concern by the World Health Organization. The disease caused by SARS-CoV-2 is termed COVID-19. It is reported that compared with SARS-CoV, although SARS-CoV-2 has lower case fatality rates (Chen et al., 2020), it has higher transmissibility and is prone to affect older patients with comorbidities (Zhang et al., 2020). From the clinical data, COVID-19 manifests with fever, non-productive cough, dyspnea, myalgia, fatigue, normal or decreased leukocyte counts, and severe lung injury (Wu et al., 2020). Severe and lethal cases also showed organ dysfunction, including shock, acute respiratory distress syndrome (ARDS), acute cardiac injury, acute kidney injury, liver dysfunction and secondary inflammation (Chen et al., 2020; Huang et al., 2020; Wang et al., 2020; Wei-jie Guan, 2020). According to the pathology of patients with COVID-19, lung tissue displays pulmonary edema and desquamation of pneumocytes and hyaline membrane formation (Ding et al., 2003; Xu et al., 2020).

A comparison of the genome of SARS-CoV-2 and SARS-CoV shows that SARS-CoV-2 has 82% nucleotide identity with SARS-CoV (Chan et al., 2020) and also used ACE2 as its receptor for entry into the cells (Wan et al., 2020). ACE2 is a carboxypeptidase catalyzing vasoactive angiotensin II (Ang II) to angiotensin-(1–7) (Ang 1-7), which acts as an antagonist of angiotensin and balances the ACE/Ang II/Ang II type I receptor (AT_1_R) axis (Richards and Raizada, 2018). Ang II via AT_1_R induces pulmonary vasoconstriction in response to hypoxia and increases vascular permeability, which results in pulmonary edema. ACE2 knockout mice exhibited more severe symptoms than control mice in an acid aspiration-induced lung injury model (Imai et al., 2005), and a recombinant form of human ACE2 is well-tolerated in patients with ARDS (Tan et al., 2018). Moreover, Ang (1–7) was found to attenuate ventilator-induced and acid aspiration-induced acute lung injury in mice (Klein et al., 2013). In summary, ACE2 plays a critical role in lung protection from injury.

Importantly, SARS-CoV infections and the SARS-Spike protein downregulates ACE2 expression (Kuba et al., 2005). Considering the homology of SARS-CoV-2 and SARS-CoV, SARS-CoV-2 may also interfere with the expression of ACE2 as well. Furthermore, it seems that this phenomenon is not unique to SARS-CoVs. The H5N1 virus-induced acute lung injury model also showed reduced ACE2 expression and increased Ang II levels (Liu et al., 2017). Given that ACE2 plays a paradoxical role in mediating viral entry and preventing tissue injury, we did functional analysis of genes co-expressed with ACE2 in lung tissue. Restoring the expression of these genes might represent a new method for the treatment of COVID-19.

Cmap is a database including gene expression profiles of various human cell lines that are exposed to different small-molecule compounds (Qu and Rajpal, 2012). Because of the expense involved in researching novel therapeutic drugs and performing long-term trials to ensure its safety and tolerance in the human body, the repurposing of known drugs is a feasible drug development strategy. Using Cmap to identify candidate drugs to treat diseases is an efficient approach. For example, valproic acid was found to have a therapeutic effect on epilepsy by using Cmap (Delahaye-Duriez et al., 2016). Trifluoperazine, as predicted by Cmap, inhibits cancer stem cell growth and overcomes drug resistance in lung cancer (Yeh et al., 2012). By uploading the query files of the DEGs and the genes co-expressed with ACE2 in lung, we identified several candidate drugs for the treatment of COVID-19.

## Materials and Methods

### Data Source

The DEGs of the RNAs isolated from the BALF of patients with COVID-19 were sourced from the previous study of Yong X et al.^1^ (Supplementary File 1). The mRNAs microarray data from five fresh healthy lung tissues are available in the GEO database. Date under accession numbers GSM4040007, GSM4040008, GSM4040009, GSM4040010, and GSM4040011, which was based on the GPL13497 (Agilent-026652 Whole Human Genome Microarray 4 × 44K v2), were contributed by Jiang N et al.

### Pathway and Process Enrichment Analysis

To investigate the main functional mechanisms of these genes, the analysis was performed using Metascape (Zhou et al., 2019) and displayed by the bubble plot using R package. To display and visualize the relationship between a list of candidate genes and terms, as well as the logFC of the genes, the GO Chord plotting function was implemented by the GO Chord package in R, and only genes that were assigned to at least one process could be displayed. The molecular functions or biological processes of ACE2 correlated gene clusters were performed by FunRich (Pathan et al., 2015).

### Construction and Analyzing of Protein-Protein-Interaction (PPI) Networks

The PPI networks with a combined score > 0. 9 were constructed by the STRING database (Version 11.0, ELIXIR, Europe, https://string-db.org/) (Szklarczyk et al., 2017). Only connected nodes were retained and analyzed by Cytoscape (Version 3.6.1, Cytoscape Consortium, U.S). Centiscape was used to calculate the degree centrality of each node (Scardoni et al., 2014). Referring to the previous study (Pang et al., 2019), we determined the nodes with degree ≥ 15 as hub genes. In order to identify densely connected network components, cluster analysis was performed by MCODE (Halary et al., 2010). Data parameter was set with thresholds of K-Cores > 5 (Luo et al., 2019).

### Analysis of Genes Co-expressed With ACE2

Results were analyzed statistically using Pearson’s correlation coefficient. The criterion was a *p*- value < 0.05. We also created statistical plots for individual genes using the R packets ggplot. All results were graphically presented in volcano plots.

### Candidate Drugs Based on Cmap Database Analysis

We uploaded files to the Cmap Web Service^2^. In the permuted results, scores ranging from -1 to 1 represented the correlation between the drug and uploading files. The more negatively correlated drugs indicated a greater correlation with the files and were more likely to be useful for the treatment.

## Results

### Functional Enrichment Analysis of the DEGs in Patients With COVID-19

**FIGURE 1:**
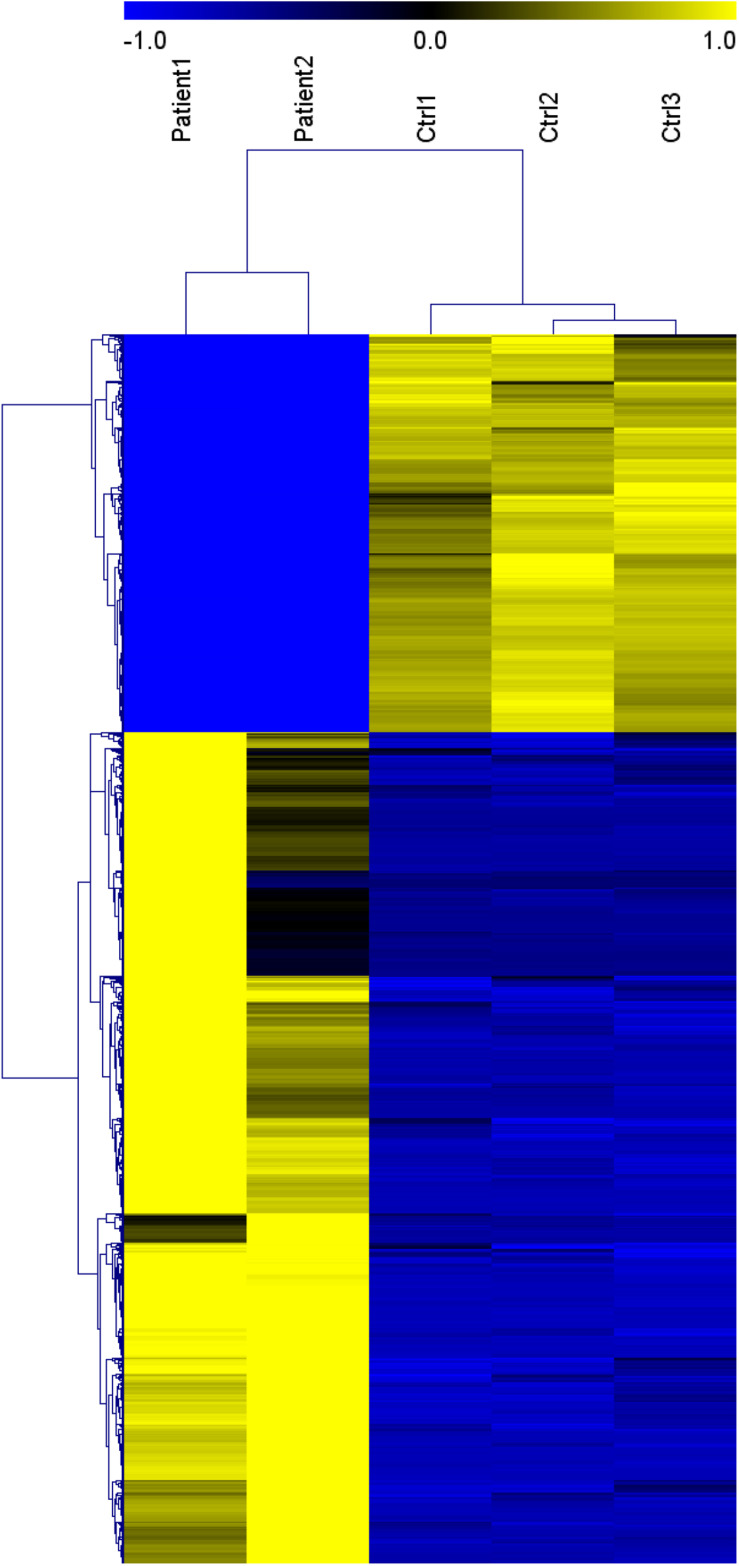
Heat map of genes significantly up-regulated and down-regulated (fold change > 2) in COVID-19 patients BALF. (Patient 1-2 vs. Ctrl 1-3). This figure is tansformed from the materials of Yong X. et al. (Xiong et al., 2020). Published by Informa UK Limited, trading as Taylor & Francis Group, on behalf of Shanghai Shangyixun Cultural Communication Co., Ltd. This is an Open Access article distributed under the terms of the Creative Commons Attribution License (http://creativecommons.org/licenses/by/4.0/), which permits unrestricted use, distribution, and reproduction in any medium, provided the original work is properly cited.

**FIGURE 2:**
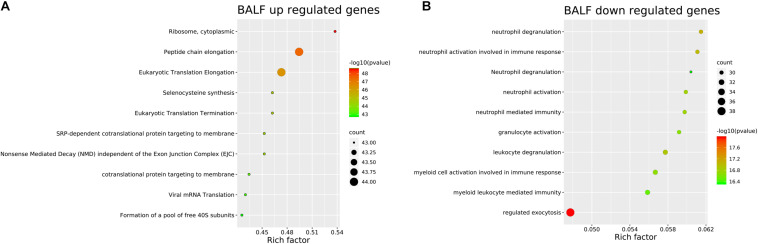
Pathway and process enrichment of DEGs in BALF of COVID-19 patients. **(A)** BALF up-regulated genes. **(B)** BALF down-regulated genes. The colors of the nodes are illustrated from red to green in descending order of -Log10 (*p* Value). The sizes of the nodes are illustrated from small to big in ascending order of gene counts. The horizontal axis represents the rich factor, the vertical axis represents the GO or KEGG terms.

Genes significantly up-regulated and down-regulated (fold change > 2) in BALF of patients with COVID-19 were identified (Xiong et al., 2020) (Supplementary File 1) and represented in the scaled heatmap (Figure 1). We performed pathway and process enrichment analysis for the DEGs. The results showed that the up-regulated genes were related to ribosome, protein translation and viral mRNA translation. The down-regulated genes were enriched in immune response such as neutrophil degranulation, neutrophil activation, granulocyte activation, leukocyte degranulation (Figure 2).

**FIGURE 3:**
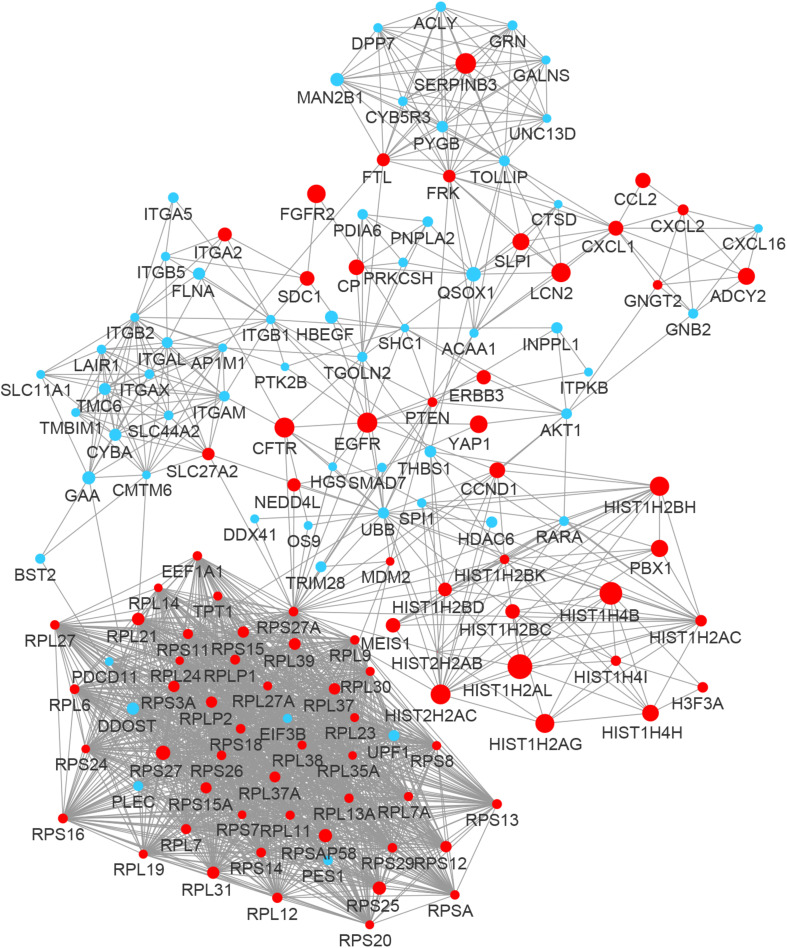
The PPI network of the DEGs in BALF of COVID-19 patients. The nodes representing up-regulated DEGs are shown as red circles and the down-regulated DEGs are presented as blue circles. The sizes of the nodes are illustrated from small to big in ascending order of |Log2 (FC)|.

**FIGURE 4:**
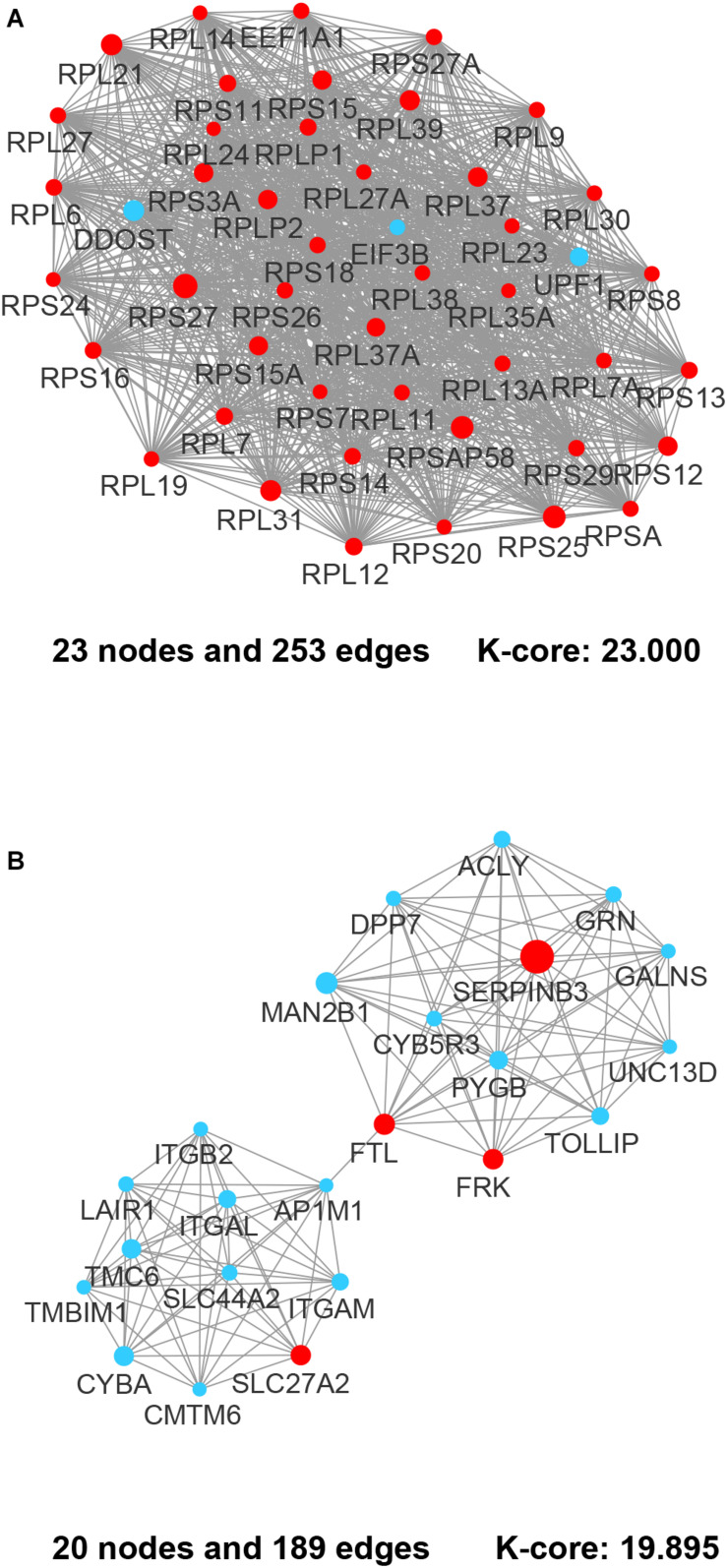
The most prominent modules of the DEGs PPI network. The nodes representing up-regulated DEGs are shown as red circles and the down-regulated DEGs are presented as blue circles. The sizes of the nodes are illustrated from small to big in ascending order of |Log2 (FC)|. **(A)** Cluster 1. **(B)** Cluster 2.

**FIGURE 5:**
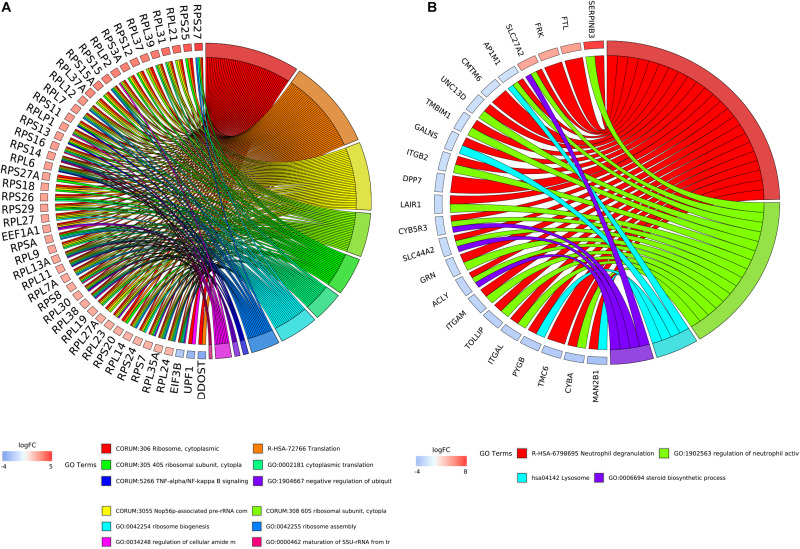
The relationship between enriched biological process terms and DEGs in clusters. **(A)** Cluster 1. **(B)** Cluster 2. The colors of the nodes are illustrated from red to blue in descending order of logFC. The genes were ordered according to their logFC values.

By integrating DEGs with a combined score > 0.9 in the STRING database and selecting hub genes whose degree ≥ 15, a PPI network of DEGs hub genes was constructed (Figure 3). In this network, DEGs that had high degree values and high absolute values of fold change, included DDOST (degree, 46; Log2(FC), −3.82), UPF1 (degree, 42; Log2(FC), −3.19), HIST2H2A (degree, 17; Log2(FC), 7.42), ITGAL (degree, 13; Log2(FC), −3.23), EGFR (degree, 13; Log2(FC), 7.52), and CXCL1 (degree, 13; Log2(FC), 5.09). To identify tightly connected regions in the network, cluster analysis was performed by MCODE. Two modules were extracted from the PPI network through MCODE analysis with K-Cores > 5 (Figure 4). Cluster 1 included genes encoding ribosomal proteins that were mostly up-regulated in patients with COVID-19. Biological pathway analysis of Cluster 2 genes showed that they were mostly related to neutrophil degranulation, regulation of neutrophil activation (Figure 5). Most of these genes were down-regulated in patients with COVID-19.

### Functional Enrichment Analysis of the Genes Co-expressed With ACE2 in Lung Tissue

**FIGURE 6:**
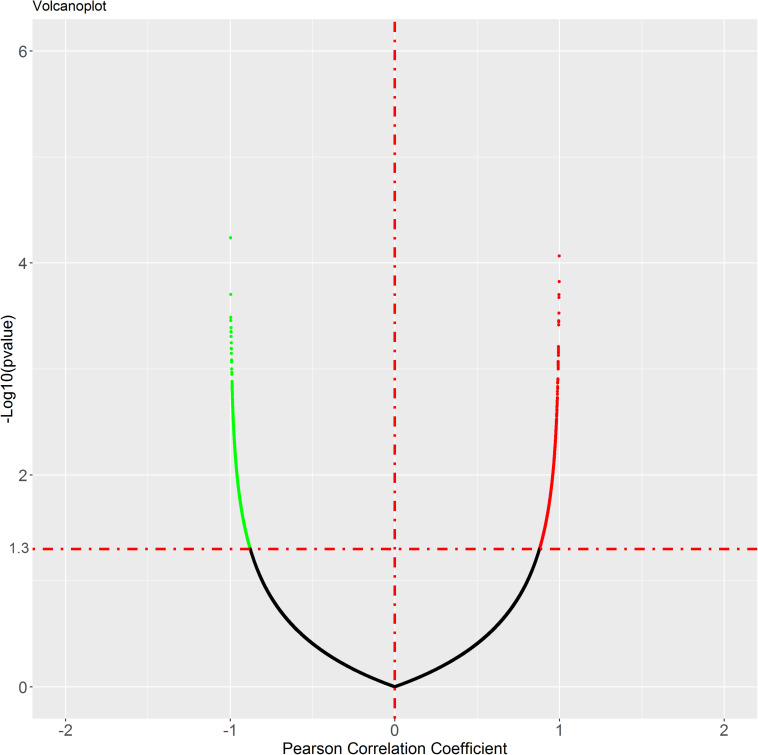
Genes correlated with ACE2 expression in lung. In the figure, black is the gene with *p* value ≥ 0.05, while green is the gene with negative correlation and red is the gene with positive correlation.

**FIGURE 7:**
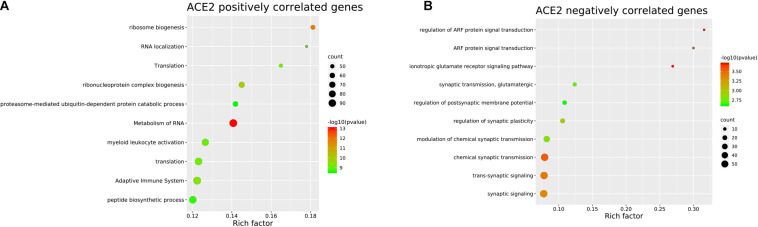
Pathway and process enrichment of the genes co-expressed with ACE2 in lung. **(A)** ACE2 positively correlated genes. **(B)** ACE2 negatively correlated genes. The colors of the nodes are illustrated from red to green in descending order of -Log10 (*p* Value). The sizes of the nodes are illustrated from small to big in ascending order of gene counts. The horizontal axis represents the rich factor, the vertical axis represents the GO or KEGG terms.

To identify the genes co-expressed with ACE2, we analyzed the co-expression of ACE2 with other genes in the normal lung tissue samples. All results were graphically presented in volcano plots (Figure 6) (Supplementary File 2). Genes with *p*-values < 0.05 were selected. Interestingly, the functional analysis results revealed that the positively correlated genes were related to metabolism of RNA, ribosome biogenesis, myeloid leukocyte activation, adaptive immune system. The negatively correlated genes were enriched in synaptic transmission and signaling functions (Figure 7).

**FIGURE 8:**
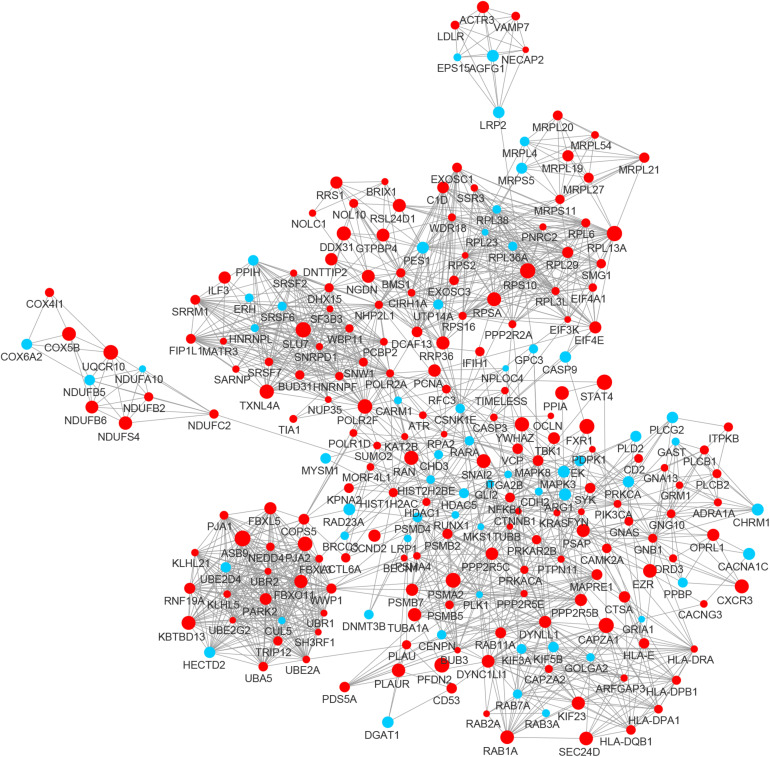
The PPI network of the genes co-expressed with ACE2 in lung. The nodes representing positively correlated genes are shown as red circles and the negatively correlated genes are presented as blue circles. The sizes of the nodes are illustrated from small to big in ascending order of correlation.

**FIGURE 9:**
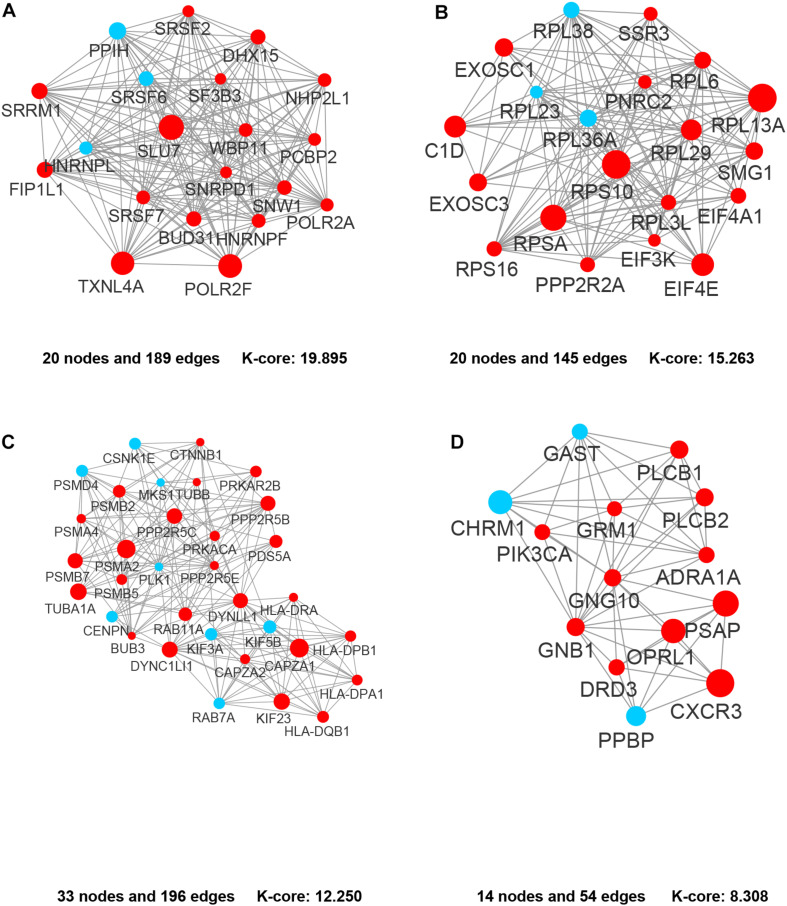
The clusters of the genes co-expressed with ACE2. **(A)** Cluster 1. **(B)** Cluster 2. **(C)** Cluster 3. **(D)** Cluster 4. The nodes representing positively correlated genes are shown as red circles and the negatively correlated genes are presented as blue circles. The sizes of the nodes are illustrated from small to big in ascending order of *r*-value.

**FIGURE 10:**
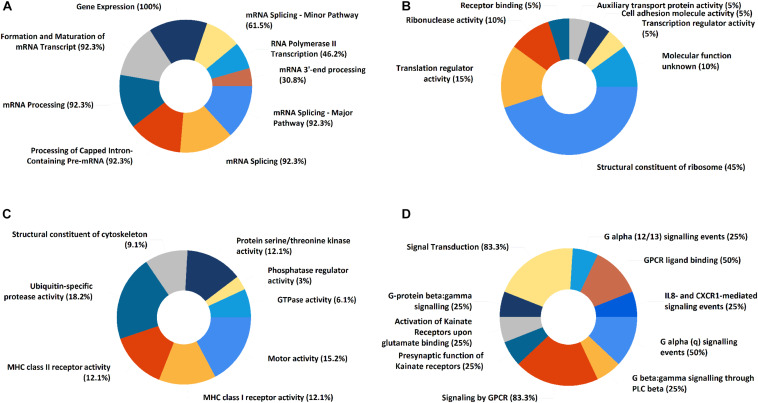
The functional enrichment of ACE2 correlated genes clusters. **(A)** Cluster 1. **(B)** Cluster 2. **(C)** Cluster 3. **(D)** Cluster 4. The numbers represent “percentage of genes.”

In the PPI network of its hub genes (Figure 8), DYNLL1 (degree, 33; r, 0.959), POLR2F (degree, 30; r, 0.985), RPL13A (degree, 25; r, 0.993), FBXO11 (degree, 23; r, 0.972), and CSNK1E (degree, 23; r, −0.932) had high degree values and were highly relevant to ACE2. Cluster analysis showed four clusters with high K-Cores (Figure 9): Cluster 1 function was mRNA processing. 45% of genes in Cluster 2 was about ribosome structure constituent. In Cluster 3, 18.2% of genes were related to ubiquitin-specific protease activity, 15.2% motor activity, 12.1% MHC class I receptor activity, 12.1% MHC class II receptor activity. The majority of Cluster 4 genes were related to G-protein-coupled receptor (GPCR) signaling (Figure 10).

### Candidate COVID-19 Drugs Predicted by Cmap

In order to identify compounds with molecular features that are capable of managing COVID-19 related symptoms, we uploaded the DEGs of the BALF into the Cmap database. Ranking based on negative connectivity scores was used to reveal the top small molecular compounds. These candidate drugs may counteract the observed gene expression pattern in the BALF of patients with COVID-19. A further eight drugs were obtained based on the genes co-expressed with ACE2 in the lung using the same method. They were speculated to reverse the expression changes in these genes when ACE2 was down-regulated following SARS-CoV-2 infection (Tables 1, 2).

**TABLE 1:** Top small molecules based on DEGs of COVID-19 patients BALF.

Rank	Cmap name	Mean	*n*	*p*-value	Specificity	Description
1	Adiphenine	−0.619	5	0.00006	0.0242	Anticholinergic drugs which can relieve intestine or stomach, palace, ureter, bile duct spasm
2	Podophyllotoxin	−0.605	4	0.0001	0.0098	A lignan found in podophyllin resin from the roots of podophyllum plants
3	Amantadine	−0.594	4	0.00068	0.0065	An antiviral that is used in the prophylactic or symptomatic treatment of influenza A. It is also used as an antiparkinsonian agent.
4	Thioperamide	−0.564	5	0.00008	0	Antifungal agents
5	Monensin	−0.542	6	0.00014	0	A polyether isolated from *Streptomyces cinnamonensis* that presents antibiotic properties
6	Vancomycin	−0.532	4	0.00066	0	Antibacterial obtained from Streptomyces orientalis
7	Etiocholanolone	−0.494	6	0.00036	0.0065	The 5-beta-reduced isomer of androsterone
8	Acyclovir	−0.416	6	0.00072	0.0066	Acyclovir is a nucleotide analog antiviral agent

*Cmap Name: the name given to a perturbation; mean: the mean connectivity score, a combination of the up score and the down score. A high negative connectivity score indicates that the corresponding perturbation conforms to the expression of the query signature; n: number of repetitive samples; *p*-value: The probability of the enrichment of a set of instances in the total set of instances by chance upon execution of a query.*

**TABLE 2:** Top small molecules based on the genes co-expressed with ACE2.

Rank	Cmap name	Mean	*n*	*p*	Specificity	Description
1	Isoflupredone	−0.754	3	0.00022	0.0083	This compound belongs to the class of organic compounds known as 21-hydroxysteroids.
2	Heptaminol	−0.647	5	0.00096	0.0137	This compound belongs to the class of organic compounds known as tertiary alcohols.
3	Chenodeoxycholic acid	−0.647	4	0.00157	0.0308	Chenodeoxycholic acid is a bile acid naturally found in the body. It works by dissolving the cholesterol that makes gallstones and inhibiting production of cholesterol in the liver and absorption in the intestines, which helps to decrease the formation of gallstones.
4	Podophyllotoxin	−0.642	4	0.00203	0.0588	The same as Table 1
5	Atractyloside	−0.609	5	0.00096	0.0076	A plant extract
6	adiphenine	−0.578	5	0.00176	0.129	The same as Table 1
7	monensin	−0.552	6	0.00028	0	the same as Table 1
8	Lisuride	−0.541	5	0.00188	0.0492	An ergot derivative that acts as an agonist at dopamine D2 receptors (dopamine agonists). It may also act as an antagonist at dopamine D1 receptors, and as an agonist at some serotonin receptors (serotonin agonists).

*Cmap Name: the name given to a perturbation; mean: the mean connectivity score, a combination of the up score and the down score. A high negative connectivity score indicates that the corresponding perturbation conforms to the expression of the query signature; n: number of repetitive samples; *p*-value: The probability of the enrichment of a set of instances in the total set of instances by chance upon execution of a query.*

## Discussion

COVID-19 became an outbreak in 2019 and continues to spread all over the world at an alarming speed. The pathogen of COVID-19 is named SARS-CoV-2, which has 82% nucleotide identity with SARS-CoV. Like SARS-CoV, SARS-CoV-2 also invades into the host by combining with ACE2. Paradoxically, ACE2 protects against lung injury in different respiratory diseases. It has been reported that SARS-CoV inhibits the expression of ACE2 in the lung after infection. The functions of the genes co-expressed with ACE2 are unclear.

By comparing the transcriptome of the BALF from patients with COVID-19 and healthy people, we found that the up-regulated DEGs were mainly concerned with protein translation and viral mRNA translation. In the stage of infections, the virus needs to usurp and redeploy host cells protein synthesis machinery including its ribosomes for translation of its own mRNA. In response, the host swift protein synthesis to antiviral stage as a strategy to limit infection damage (Hoang et al., 2018). The down-regulated DEGs were related to immune cell degranulation and activation, which lead to immune dysfunction. According to the MCODE analysis, the clusters also mainly involved in ribosome constituent and neutrophil immune response.

Using genome-wide RNA-sequencing data of healthy lung tissues, we found 1580 positively correlated and 1282 negatively correlated genes of ACE2. Genes positively correlated with ACE2 regulated protein translation, myeloid leukocyte activation, and adaptive immune system. It is an effective strategy for virus to inhibit ACE2 as well as those positively correlated genes for escaping from immune surveillance. The negatively correlated genes were involved with synaptic transmission and signaling. ACE2 overexpression in the brain attenuates the enhanced cholinergic synaptic transmission in spontaneously hypertensive rats (Deng et al., 2019). Therefore, the mechanism of ACE2 in attenuating vasoconstriction may not only involve the conversion of Ang II to Ang (1–7), but also the inhibition synaptic transmission.

Cluster analysis revealed that these gene clusters were mainly about mRNA processing, ribosome structure constituents, MHC class I and II receptor activity, GPCR signaling. GPCR signaling is essential for the spatiotemporal control of leukocyte dynamics during immune responses (Lammermann and Kastenmuller, 2019). A recent study analyzing the genes co-expressed with ACE2 in colonic epithelial cells reported that they were enriched in viral infection and egress, innate immune responses, inflammation and apoptosis (Jun Wang et al., 2020). From the above, genes co-expressed with ACE2 are associated with ribosome assembly and immune response.

Next, we found out several candidate drugs for COVID-19 using Cmap based on the DEGs and the genes co-expressed with ACE2. Many successful applications of drug repurposing have been reported using the above strategy, such as cancer (Sirota et al., 2011), muscle atrophy (Kunkel et al., 2011), acute myelogenous leukemia (Hassane et al., 2008). Candidate therapeutic molecules are listed in Tables 1, 2. These drugs are speculated to counteract the altered gene expression in the BALF of patients with COVID-19, or reverse gene transcriptional changes when ACE2 is down-regulated following infection. Tables 1, 2 shows common results including podophyllotoxin, adiphenine, and monensin with greater probabilities of curing the disease. Podophyllotoxin is highly active against HIV and human papillomavirus (HPV) *in vitro* (Hensel et al., 2020). Amantadine and acyclovir, as antiviral agents, are also predicted to treat COVID-19, and amantadine has shown therapeutic effects in other studies on COVID-19 treatment (Aranda Abreu et al., 2020; Brenner, 2020; Rejdak and Grieb, 2020). However, the therapeutic effects of candidate drugs identified by Cmap predictions must be further investigated to generate empirical evidence.

## Conclusion

We utilized the gene expression profiles of BALF in patients with COVID-19 and found that the DEGs were associated with ribosome constituent and immune response. In addition, we found that the genes co-expressed with ACE2 in the lung mainly functioned in protein translation, immune response and synaptic transmission. Importantly, ACE2 is down-regulated in SARS-CoV or H5N1 infection. It is not only a direct access for SARS-CoV-2 invasion but also a protective molecule. Amantadine, acyclovir, podophyllotoxin, adiphenine, and monensin were candidate drugs for COVID-19 treatment according to Cmap prediction. These results provided a firm foundation for further *in vitro* and *in vivo* research regarding COVID-19 drug treatment.

## Data Availability Statement

All datasets presented in this study are included in the article/Supplementary Material.

## Author Contributions

ZL performed the experiments and drafted the manuscript. LY was responsible for the concept, study design and revised the manuscript.

## Conflict of Interest

The authors declare that the research was conducted in the absence of any commercial or financial relationships that could be construed as a potential conflict of interest.

## Footnotes

1 https://www.tandfonline.com/doi/full/10.1080/22221751.2020.1747363

2 https://portals.broadinstitute.org/Cmap/index.jsp

## Abbreviations

COVID-19: coronavirus disease 2019
Cmap: the connectivity map
SARS-CoV-2: severe acute respiratory syndrome coronavirus 2
ACE2: angiotensin-converting enzyme 2
ARDS: acute respiratory distress syndrome
DEGs: the differentially expressed genes
BALF: bronchoalveolar lavage fluid
Ang II: angiotensin II
Ang (1-7): angiotensin-(1–7)
AT_1_R: Ang II type I receptor
PPI: protein-protein-interaction
GPCR: G-protein -coupled receptor
HPV: human papillomavirus.
